# From Qualitative to Quantitative Functional Assessment in Stroke Rehabilitation with a Focus on Ultrasound Role

**DOI:** 10.3390/biomedicines13112594

**Published:** 2025-10-23

**Authors:** Rosita Rabbito, Eleonora Ficiarà, Lorenzo Priano, Matteo Bigoni, Caterina Guiot, Silvestro Roatta

**Affiliations:** 1 Department of Neurosciences “Rita Levi Montalcini”, University of Turin, 10125 Torino, TO, Italy; lorenzo.priano@unito.it (L.P.); caterina.guiot@unito.it (C.G.); silvestro.roatta@unito.it (S.R.); 2 School of Pharmacy, University of Camerino, 62032 Camerino, MC, Italy; eleonora.ficiara@unicam.it; 3 Neurology and Neurorehabilitation Unit, IRCCS Istituto Auxologico Italiano, San Giuseppe Hospital, 28824 Piancavallo, VB, Italy; m.bigoni@auxologico.it

**Keywords:** stroke, rehabilitation, assessment, scale, quantitative, ultrasound

## Abstract

Stroke-surviving patients may present a wide range of neurological deficits affecting both sensory and motor functions as well as the cognitive and the emotional domains, with an impact on independence on daily activities and quality of life in general. Assessment scales are essential tools for evaluating all these aspects of a patient’s condition and for monitoring their evolution in time, attempting to provide a quantitative index to complex and sometimes indirectly observable parameters. In fact, the use of these scales entails methodological and interpretative challenges that can limit their applicability and effectiveness. This narrative review explores the current state and limitations of assessment scales used in the rehabilitative evaluation of post-stroke patients. Common neurorehabilitation techniques and traditionally used assessment scales for measuring patient progress are reviewed, highlighting their main limitations. As an alternative to the observational approach, direct assessment of the effect of the ongoing rehabilitative process on the functional recovery of the damaged neurological network, based on the recording of their electric signaling or on the modification in regional cerebral blood flow, have been recently proposed. Innovative rehabilitation assessment methods based on quantitative data are reviewed, with a special focus on ultrasound-based techniques, aiming to improve accuracy and sensitivity in clinical assessment.

## 1. Introduction

In Italy, strokes are responsible for 9–10% of all deaths, representing the second cause of death and the first cause of disability. It is important to underline that only 25% of patients who survive a stroke recover completely, the remainder survive with some degree of disability and at least one half lose self-sufficiency in the activities of daily living (ADL) [1].

The term “stroke”, or ictus in Latin, broadly refers to a cerebrovascular event affecting the central nervous system, including both ischemic and hemorrhagic types. Clinically, an infarction specifically describes the ischemic mechanism, where insufficient blood flow (ischemia), due to an occlusion, leads to the death of a portion of brain, spinal cord, or retinal cells [2]. Instead, a cerebrovascular accident occurs by a hemorrhagic event when the blood vessels either within the brain or on its surface rupture [3]. Although age is a major risk factor, strokes can occur at any stage of life and may be associated with various underlying conditions such as cardiovascular disease, trauma, infections, malignancies, vascular malformations, autoimmune disorders, or exposure to toxins [4,5].

Stroke rehabilitation has made significant progress in the last decades, but a major challenge remains: the way we assess recovery is still limited, often based on subjective measures that may not fully capture the complexity of the healing process. This narrative article aims to discuss this limitation and explore possible future paths, integrating clinical experience with research insights. Indeed, although stroke rehabilitation has made substantial progress, with increasingly sophisticated therapeutic options that help treat motor, cognitive and sensory deficits, it still often relies on clinical scales and qualitative observations for the assessment of patient recovery. Consequently, there is a growing interest in tools that can provide objective, real-time and meaningful data to support decision-making throughout the rehabilitation process. In this context, ultrasound (US) techniques and modalities could be promising tools to bridge the gap between qualitative and quantitative assessments. Being non-invasive, widely accessible, portable and relatively inexpensive, ultrasound imaging represents a strong candidate for integration into routine stroke rehabilitation. US could be considered not only as a diagnostic tool, but also as a source of biomarkers or functional indices. It is therefore one of the objectives of this narrative review to explore and define the emerging role of US in the post-stroke rehabilitation landscape. Recent technological advances have expanded the application of ultrasound for quantitative and functional assessment in post-stroke rehabilitation. Ultrasound-based modalities can provide quantitative data regarding the motor component, such as elastography, which can quantify muscle stiffness and spasticity, or quantitative ultrasound (QUS) techniques, which include analysis of echogenicity and muscle elasticity [6,7]. At the cerebral level, transcranial Doppler (TCD) and ultrasound perfusion imaging provide complementary information on cerebrovascular function, also integrating biofeedback paradigms useful for neuromodulation and neuroplasticity [8,9].

These diverse applications highlight the versatility of ultrasound as a quantitative, functional, and potentially interventional tool, strengthening its role in bridging the gap between qualitative and quantitative assessment in stroke rehabilitation.

To achieve this, a literature search was conducted in PubMed, Scopus, and Web of Science, focusing on publications from 2000 to the present. The literature was screened to identify both clinical and experimental contributions addressing the transition from qualitative to quantitative approaches in stroke recovery assessment.

Section 2 outlines the current neurorehabilitation techniques available in clinical settings. These range from well-established traditional approaches to newer, more innovative strategies. Each method addresses different aspects of recovery and reflects the growing understanding that stroke rehabilitation must be tailored to the specific needs of each individual (motor, sensory, or cognitive). Section 3 focuses on the assessment tools most widely used to evaluate patient progress. These clinical scales are essential for quantifying impairments and tracking outcomes, yet they often provide only a partial picture. We discuss their benefits and limitations, especially the risk of overlooking subtle or dynamic aspects of recovery when relying solely on these tools. Section 4 highlights innovative and research-oriented approaches that bring an objective and real-time dimension to rehabilitation. Ultrasound-based methods exemplify the translational potential of technologies capable of integrating scientific insight with clinical practice.

## 2. Neurorehabilitation: Principles and Techniques

Neurorehabilitation plays a pivotal role, particularly for stroke survivors, where over a third continue to experience significant disability years after the event, preventing them from achieving independence. In the central nervous system, the regenerative potential of neural tissue is limited, making spontaneous recovery generally insufficient. However, cerebral recovery after injury can be significantly influenced by external stimuli and interaction with the environment. Through targeted rehabilitation processes, it becomes possible to promote neural recovery and functional improvement.

This recovery occurs through the brain’s ability to reorganize and compensate for the damage caused by the stroke. The key mechanism is neuroplasticity, where undamaged areas of the brain take over functions previously controlled by the damaged regions, or recruit alternative neural pathways through a reorganization of cortical networks. Peripheral stimulations, facilitated through various therapeutic interventions, help to activate these residual central mechanisms [10]. The recovery of movement and sensation is often supported by improved blood flow through collateral circulation, which helps to restore metabolic activity in affected areas that were not entirely destroyed by the stroke. Such blood flow is also used in functional magnetic resonance (fMRI) studies as a marker of this cortical activation and would be interesting to find new other techniques able to evidence such variation.

Rehabilitation techniques generally fall into two main categories: neurofacilitation, which aims to enhance the nervous system’s natural capacity for recovery; and compensation, where strategies are developed to adapt to lasting impairments. In both traditional and more modern rehabilitation approaches these two milestones can be found [11]. The first one, also named Proprioceptive Neuromuscular Facilitation (PNF), leverages the body’s proprioceptive system to stimulate neural pathways and promote activity-dependent neuroplasticity, as the brain’s ability to reorganize and form new connections in response to targeted stimulation [12]. The second one is aimed at supporting the patient’s activity and participation with strategies and tools to overcome functional limitations. With both it is possible to exploit the task-oriented approach, which is a fundamental method of presenting rehabilitation interventions, inserted in an ecological context, and which allows to leverage the patient’s motivation [13]. The rationale of these methods can be applied in every rehabilitation area such as: sensorimotor deficits, speech and swallowing disorders, cognitive disorders [14].

When addressing post-stroke recovery of motor functions, the traditional approach is typically administered through physiotherapy sessions. Normally, the rehabilitation team (physical therapists in particular) follow different methods developed mainly in the second half of the twentieth century. Even before the discovery of neuroplasticity, rehabilitation techniques with organized treatment schemes were developed. The elements that characterize and differentiate the treatments derive from distinct interpretative theories regarding the functioning of the nervous system and its recovery mechanisms. Among the different rehabilitative techniques, as example, some of the most widespread interventions can be considered: the Cognitive Therapeutic Exercise (ETC) by Perfetti [15] and the Bobath method [16,17], have long been applied to promote motor recovery after stroke, focusing respectively on the integration of sensory and motor feedback and on facilitating neuroplasticity to restore functional movement [18,19].

Contemporary therapeutic strategies have embraced innovative methodologies designed to promote cerebral recovery through non-motor stimuli. Techniques such as brain stimulation, mirror therapy, and mental practice leverage advancements in neurophysiology and neuropathology, particularly in understanding neural connectivity and plasticity. These approaches stimulate recovery by enhancing the brain’s natural mechanisms for reorganization without direct physical activity.

Modern strategies are often integrated with instrumental neuromodulation techniques that utilize physical modalities like electricity and magnetism. Additionally, robotic-assisted devices are widely employed for targeted motor rehabilitation of limbs and gait. Virtual reality-based interventions, including exergames, offer immersive environments that engage patients in interactive and adaptive rehabilitation exercises. These tools not only support physical recovery but also extend the scope of neurorehabilitation, allowing for personalized and accessible treatment options.

Table 1 below summarizes contemporary approaches and technologies in post-stroke rehabilitation. The table integrates both conventional and technology-driven strategies, emphasizing their underlying mechanisms and current utilization status. While conventional clinical protocols and robotic or telemedicine-based systems are already well integrated into routine rehabilitation practice, neuromodulation-based techniques remain at an experimental or early clinical stage despite promising evidence accumulated over the past two decades.

## 3. Evaluating Stroke Impact: Insights into Assessment Scales in Neurorehabilitation

Stroke is a highly debilitating disorder that unquestionably necessitates specific neurorehabilitation. The central core of any rehabilitation process is undoubtedly focused on reducing the disability and enhancing the functional independence of the patient. Indeed, it is estimated that 60% of the stroke survivors can regain independence, with 75% achieving autonomous walking [35].

The essential preliminary data for properly designing a rehabilitative intervention includes the location and extent of the lesion, the individual’s age and medical history, assessment of brain and motor function, as well as of personal and environmental factors. Furthermore, such data allows for a comparison of the effectiveness of various rehabilitation methodologies and for an objective and measurable quantification of the progress achieved during the treatment.

Currently, many scales have been proposed for assessing rehabilitation effectiveness but a universally ideal scale for evaluating stroke survivors is still missing. The choice of the best scale or scales should, therefore, be guided by the specific aspects or parameters to be assessed. Above this, to achieve a more comprehensive overview of the patient, a very popular approach is the use of International Classification of Functioning, Disability, and Health (ICF). This scale was introduced in 1999 by the World Health Organization (WHO) [36] and provides a basis for describing the macro-categories body function, activities, and social participation, offering a comprehensive structured framework.

The ICF-oriented approach, overcoming the simple analysis of individual deficits or disability, focuses on the intrinsic capacities of the individual, accounting for the dynamic relationship between disability, activities, and participation, also shaped by personal and environmental factors. Several studies substantiate the utility of the ICF in research [37,38,39], providing an overall prediction of the outcome of the treatment.

A list of the systematic approaches is given in Supplementary Section S1, outlining the most widely employed assessment scales in both clinical and research settings organized in accordance with the ICF structure, describing functioning and disability.

### 3.1. Challenges and the Quest for Objectivity

Different clinical scales are designed to assess specific aspects of a patient’s condition and rehabilitation outcomes. However, the complexity of clinical cases often requires the use of multiple scales to obtain a comprehensive view. To achieve a more accurate and holistic assessment, it is therefore crucial to adopt tools that integrate multiple domains. However, the choice of such tools varies considerably across medical institutions and research settings. The lack of a standard means that clinical and scientific practices are largely shaped by the specific needs of each context and represent a major limitation to the comparability of results between studies and clinical centers, ultimately hindering the generalization and translation of results into broader clinical practice.

This diversity of approaches and the ongoing need to broaden the complexity of clinical assessments underscore the importance of carefully considering instrumental choices based on the specific needs and characteristics of each case.

### 3.2. Limitations of the Use of Single Scales

Several studies highlighted the relevance of the Barthel Index (BI) [40] as a measure of the effectiveness of rehabilitative therapies, in assessing specific daily activities and physiological deficits [41,42]. However, it is important to note that BI has significant limitations due to the lack of consideration for crucial aspects of personal independence, such as cognition, language, visual function, and pain. These elements are of fundamental importance in the overall assessment of disability and are not adequately reflected by the BI score. Additionally, the presence of floor and ceiling effects further undermines the reliability of the scale [43,44], inadequate to describe patient conditions at the boundaries of the range.

This challenge extends beyond the BI and is observed in other scales, like the Functional Independence Measure (FIM) [45,46], further stressing the need for assessment tools both more sensitive and comprehensive.

### 3.3. Non-Linear Dynamics and Redundancy

Contrary to a linear progression, assessment scales exhibit a nuanced structure where advancement through each step does not translate in a proportional increase in ability [47]. The possible bias arises because some steps may be more challenging to overcome than others and surpassing one step does not necessarily imply a corresponding increase in ability. Another obstacle to linearity is redundancy in items. In situations where different scale items describe distinct behaviors but reflect the same level of ability, a redundancy issue occurs. If a subject surpasses one of these items, they are likely to surpass the others as well, leading to an overestimation of the overall variation. This phenomenon, known as “inflation”, can compromise the accuracy of the assessment, generating overly optimistic estimates of the improvement.

### 3.4. Reproducibility, External Variables, and Differential Item Functioning

When using these clinical questionnaires, it is essential to consider the issue of measurement reproducibility [47], as external or confounding factors, such as emotional state, social context, or environmental stressors, can affect patient responses independently of their actual clinical condition. These influences introduce variability that may compromise the validity and reliability of results. Although it is often impossible to eliminate such factors entirely, their monitoring and the transparent reporting of testing conditions, along with strategies to minimize their impact, are essential to ensure the accuracy of clinical assessments. “Differential item functioning” (DIF) significantly influences the use of assessment scales by introducing another concern about variability related to how specific items discriminate across diverse groups of individuals, despite their possessing the same overall ability as measured by the scale [48,49]. For instance, using an assessment scale to measure linguistic ability, some items could make use of words or concepts that can be interpreted differently among people from different cultures or backgrounds. If these differences in interpretation influence the responses of individuals of equal linguistic competence, DIF occurs. DIF can compromise the comparability of scores between subgroups, as the obtained scores may reflect not only actual differences in the abilities measured by the scale but also influences due to items with DIF. This phenomenon makes it challenging to interpret assessment results accurately and can lead to erroneous conclusions based on the obtained scores.

The recognition that assessment scale scores do not represent true, continuous, or linear measures stems from the inherent complexity of clinical evaluations. These scores should not be viewed as precise, objective metrics of an individual’s abilities or conditions but as indicators that can help the medical evaluation.

### 3.5. Inter-Observer Variability

Further support for this careful management of the clinical scales comes from the observation that final scores on a scale can vary significantly depending on the assessor. This inter-observer variability affects the subjective dimension of the assessment, indicating that there is no absolute standardization in the perception and assignment of scores. Therefore, the evaluation is a qualitative one, capturing nuanced and not easily quantifiable aspects, but also subjective, reflecting the individual interpretation and preferences of the assessor. This subjectivity can arise from various factors, including personal experiences, assessment styles, and interpretations of scale items. Moreover, the specific context in which the assessment takes place can significantly influence the final scores. This understanding of the raw, qualitative, and subjective nature of assessment scale scores highlights the complexity and challenges associated with interpreting their results in a clinical setting.

Another difficulty encountered in the clinical setting is the final robustness of the results achieved. This challenge derives from the complexity of patient-provider interactions, the individual peculiarities of the patient, the fluctuating health status, the wide range of applicable treatments, and the limited repeatability of measures obtained through the use of assessment scales and clinical tests [50]. The uniqueness of the patient, coupled with the range of different available treatments, contributes to a constantly evolving clinical landscape, adding further complexity to the final assessment. The poor reproducibility of the measures obtained with assessment scales and clinical tests further impact the consistency and reliability of the results over time, and can be attributed to various sources, including natural fluctuations in the patient’s condition, differences in assessor interpretations, and environmental influences.

Over the past decade, researchers and clinicians in rehabilitation have shifted toward more reliable assessment methodologies that incorporate quantitative indicators. This approach aims to enhance the robustness of evaluations through numerical data while preserving the richness of qualitative insights [51].

In conclusion, the quest for more reliable and balanced approaches that account for the qualitative and quantitative complexities of clinical conditions is a fundamental step toward enhancing the accuracy and objectivity of clinical assessments. However, factors such as non-linear dynamics, redundancy, reproducibility, external variables, differential item functioning, and inter-observer variability can significantly influence the objectivity of these measures, potentially limiting the consistency and validity of clinical evaluations.

## 4. Towards Quantitative Assessment of Stroke Impact: Role of Ultrasound Methodologies

**Figure 1:**
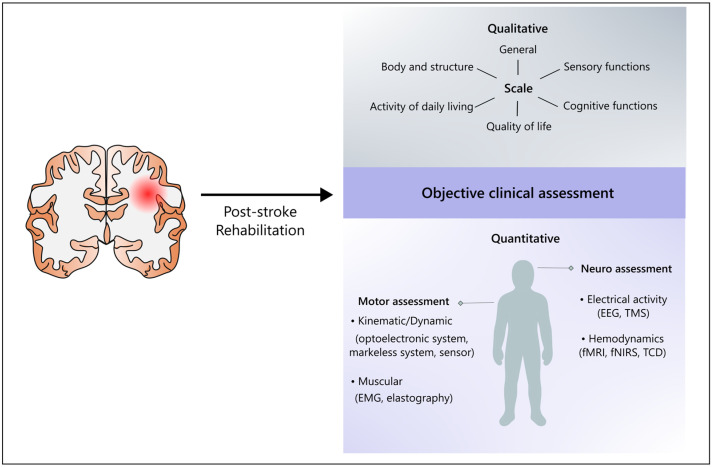
Post-stroke rehabilitation requires a multidimensional assessment approach that integrates both qualitative and quantitative evaluations. Traditional qualitative scales assess aspects like body structure, sensory and cognitive functions, and quality of life but have limitations in sensitivity and objectivity. In contrast, quantitative methods offer more precise insights into motor and neural function recovery. Combining these tools enhances monitoring and personalization of therapy, supporting a more tailored and effective rehabilitation pathway.

In addition to traditional clinical scales, several quantitative methodologies have been developed to objectively assess rehabilitation outcomes in post-stroke patients (see Figure 1). These approaches can be broadly divided into two main categories: those aimed at assessing motor control, and those focused on evaluating neurovascular and neurofunctional responses.

### 4.1. Assessment of Motor Function

One of the most common deficits following stroke involves motor function, whether in terms of strength, coordination, or movement itself. Over the years, various technologies have been developed to monitor and improve patients’ motor function. These approaches often focus on monitoring improvements in kinematic and dynamic parameters, offering detailed insights into motor recovery. A well-established method for tracking motor improvement in stroke rehabilitation involves optoelectronic systems. These systems, which use cameras and markers, analyze patient movement during rehabilitation sessions to evaluate progress or compare therapeutic interventions between patient groups. For example, Razfar et al. [52] employed wearable and camera-based sensors to automate stroke severity assessment, while Chen et al. [53] described kinematic parameters during upper-limb tasks related to daily living activities, providing valuable data for tracking motor improvements. Similarly, Krawczyk et al. [54] compared physiotherapeutic approaches using kinematic analysis for gain improvement in sub-acute stroke patients, and Titus et al. [55] analyzed trunk kinematics and gait parameters to assess recovery during stroke rehabilitation.

Recent years have seen advancements in markerless systems and wearable sensor technologies, offering cost-effective and user-friendly alternatives to traditional setups. Markerless systems, such as those utilizing Azure Kinect or smartphone-based pose estimation, capture motion data without requiring physical markers, enhancing accessibility while maintaining accuracy [56,57]. Wearable sensors, such as the BEAGLE kinematic system described by Malesevic et al. [58], enable continuous monitoring of movement and have been used to assess robotic-assisted rehabilitation and hand function. Game-based technologies like the Xbox Kinect have also been explored for rehabilitation, with studies like Schaham et al. [59] demonstrating their effectiveness in stroke therapy. For instance, Nayeem et al. [60] introduced the “MAGIC Table”, a custom-designed device equipped with a high-resolution camera capable of real-time kinematic recording. The device allowed for quantitative comparison of stroke patients and healthy subjects in their interaction with various objects.

Beyond motion analysis, innovative methodologies leverage artificial intelligence (AI) and machine learning (ML) to predict rehabilitation outcomes. For example, Bai et al. [61] investigated a spatiotemporal graph convolutional networks based on accurate posture measurements acquired by two Azure Kinect devices for upper limb rehabilitation assessment. Similarly, Yamamoto et al. [62] mapped the finger movements via wearable devices and employed AI tools as indicated by Lu et al. [63]. Such tools encompass the use of sensors, which were proposed by other authors, for instance, to detect force feedback [64], or depth [65]. In fact, Campagnini et al. [66] highlighted the potential of ML to integrate kinematic and clinical data for recovery prognosis. These approaches offer the potential for personalized rehabilitation programs by identifying patient-specific recovery patterns.

Another technique for monitoring motor function is certainly electromyography (EMG); it is based on recording the electrical signals generated by muscles and has a significant role in stroke rehabilitation because it provides important information on muscle activity and can quantify muscle function including muscle weakness, spasticity and impaired coordination of movements. This information is valuable for assessing motor disorders, monitoring recovery and guiding rehabilitation strategies [67,68,69,70].

An emerging and promising approach in the quantitative assessment of post-stroke motor impairment involves the use of ultrasound imaging, particularly ultrasound elastography, to characterize the mechanical properties of muscle tissue. Unlike traditional clinical scales (see table in Supplementary Material, BODY AND STRUCTURE section), which are qualitative and suffer from limited reliability, elastography techniques provide objective, reproducible, and quantitative metrics of muscle stiffness, allowing for a more precise evaluation of spasticity and motor recovery [71]. Two main elastography modalities are currently applied in the post-stroke setting: strain elastography and shear wave elastography [6]. These techniques have been employed to assess changes in stiffness in several muscle groups commonly affected by stroke and, typically, the paretic side demonstrates significantly increased stiffness compared to the non-paretic side [72]. A recent systematic review by Roots et al. [6] confirmed that elastography techniques consistently detect increased muscle stiffness on the paretic side. The review also noted that interventions such as passive stretching or voluntary activation further increase stiffness, suggesting that ultrasound elastography can sensitively track changes related to therapy and neuromuscular state. Beyond passive assessment, elastography has also been combined with intervention monitoring. For example, sonoelastographic evaluation has been used to quantify changes in upper limb muscle stiffness before and after kinesiotaping, demonstrating its utility in monitoring therapeutic outcomes [73]. In a different application, Tran et al. [7] discussed how QUS techniques such as pixel intensity and ultrasound strain imaging can also be used to assess post-stroke muscle echogenicity and elasticity. Furthermore, ultrasound techniques can be integrated with other approaches, as demonstrated by Cao et al. [74], who proposed a wearable system combining functional electrical stimulation and musculoskeletal ultrasound to improve intention recognition and support motor-functional recovery.

### 4.2. Assessment of Brain Function

In addition to motor assessment, understanding and assessing brain function is very important especially in the context of understanding and monitoring post-stroke recovery. Monitoring the brain response to rehabilitation can be performed through two main physiological domains: electrical activity and hemodynamic activity. Electrical monitoring techniques focus on capturing neural signals generated by neurons in the brain. These include electroencephalography (EEG) [75,76,77,78,79,80] and evoked potentials obtained by transcranial magnetic stimulation (TMS) [81], which can detect changes in neuronal responsiveness to motor or cognitive tasks and can provide indirect information on cortical plasticity and functional reorganization. Although these tools offer valuable temporal resolution and are widely used in research, their clinical application often requires a complex setup, high sensitivity to artifacts, and specialized interpretation, which may limit their widespread use in clinical settings.

Another very promising approach is based on the investigation of cerebral hemodynamics. Cerebral blood flow (CBF) is regulated by a combination of several factors, including neural, myogenic and metabolic mechanisms, and is influenced by both spontaneous and induced changes in arterial blood pressure. A key concept in this context is ‘neurovascular coupling’ that reflects the close temporal and regional linkage between neural activity and cerebral blood flow [82]. This mechanism ensures that blood flow is dynamically adjusted to meet the energy-demand of brain processes, ensuring adequate supply of oxygen and nutrients during cognitive and motor activities [83]. Monitoring the hemodynamic changes taking place during these tasks is crucial, as may reveal alteration of the neurovascular coupling following stroke or traumatic brain injuries. In the case of stroke, a compromised neurovascular coupling is observed, particularly in the affected hemisphere, due to cerebral edema, inflammation, compromised neurotransmission, and neuronal death, or to the activation of non-specific brain structures [82].

Another very promising approach is based on the investigation of cerebral hemodynamics. Cerebral blood flow (CBF) is regulated by a combination of several factors, including neural, myogenic and metabolic mechanisms, and is influenced by both spontaneous and induced changes in arterial blood pressure. A key concept in this context is ‘neurovascular coupling’ that reflects the close temporal and regional linkage between neural activity and cerebral blood flow [82]. This mechanism ensures that blood flow is dynamically adjusted to meet the energy-demand of brain processes, ensuring adequate supply of oxygen and nutrients during cognitive and motor activities [83]. Monitoring the hemodynamic changes taking place during these tasks is crucial, as may reveal alteration of the neurovascular coupling following stroke or traumatic brain injuries. In the case of stroke, a compromised neurovascular coupling is observed, particularly in the affected hemisphere, due to cerebral edema, inflammation, compromised neurotransmission, and neuronal death, or to the activation of non-specific brain structures [82].

Neuroimaging techniques, including fMRI and functional near-infrared spectroscopy (fNIRS), have proven valuable in assessing these changes. For example, fMRI studies have demonstrated cortical reorganization and motor network recovery after targeted rehabilitation programs [84,85]. Similarly, fNIRS has been used to monitor cortical activation during passive and active limb rehabilitation, providing insights into optimal therapeutic protocols [86,87].

A further methodology for investigating cerebral hemodynamics is offered by Functional transcranial Doppler (fTCD). By capturing local functional hyperemia, fTCD assesses the relationship between blood flow in the large cerebral arteries and the metabolic demands of the supplied cerebral areas, which can be modulated by activations of neural networks during specific tasks activities proposed to the patient. Tailored lateralized tasks that preferentially activate one hemisphere can help identify interhemispheric imbalances in stroke patients, evaluate rehabilitation effectiveness, and track recovery progress. Studies have shown that the preservation of functional activity in damaged brain regions is critical for clinical recovery, even in the presence of structural lesions [8,88]. For example, movement-restriction therapies have been associated with improved cerebral perfusion and enhanced hemodynamic responses [89,90]. Furthermore, biofeedback and physical exercise protocols leveraging the self-regulatory capacity of cerebral blood flow have opened new therapeutic perspectives [9,91].

In a previous study TCD was used to monitor vascular responses during highly lateralized tasks in healthy individuals [92]. Selective visual stimuli from the left and right visual hemifield were shown to evoke pronounced hyperemic responses in the contralateral posterior cerebral artery, as expected from the splitting of retinal afferent pathways at the optic chiasm. These findings provide a physiological model for monitoring post-stroke recovery in cases of hemianopsia and for developing new rehabilitative methodologies. In subsequent research, hemodynamic responses elicited by real and imagined motor tasks were investigated with the purpose of defining protocols for patient application [93]. Active tasks elicited significant contralateral hyperemia, particularly in the left-sided tasks, while imagined tasks mostly resulted in bilateral responses, highlighting the need for task-specific protocols [94,95]. Studies such as Venkatakrishnan et al. [96] and Treger et al. [97] demonstrate that TCD-derived metrics, particularly about brain lateralization and mean flow velocity in the brain arteries, can predict functional recovery and correlate with motor and neurological scales. Additionally, using these ultrasound tools, a TCD-based neurofeedback system has been developed to monitor interhemispheric hemodynamic imbalances by measuring bilateral middle cerebral artery velocities [98]. This methodology may provide a cost-effective and accessible alternative to the traditional neurofeedback system and introduce new applications in neurorehabilitation to stimulate targeted brain regions, improve cerebral circulation and neuroplasticity [86,99].

Advancements have also extended the role of ultrasound beyond the assessment of vessel patency. For example, TCD waveform morphology can aid in the detection and monitoring of large vessel occlusions, as highlighted in the review by Dorn et al. [100], contributing to improved acute stroke triage and outcome prediction. Moreover, multimodal ultrasound techniques offer valuable information on plaque vulnerability and support stroke risk stratification [101].

In recent years, other ultrasound-based techniques have broadened the scope of neurorehabilitation research and offer the possibility to observe neurovascular and neuronal processes. Among these, micro-ultrasound stands out for its potential in monitoring brain recovery mechanisms, as demonstrated by the studies of Yu et al. [102] on neurovascular remodeling in post-rehabilitation animal models.

## 5. Conclusions

The clinical assessment of post-stroke recovery is a complex challenge that requires a balance between qualitative and quantitative approaches. Traditional scales, though widely used, have limitations in terms of objectivity and sensitivity to specific changes in the patient’s condition. Quantitative methods provide crucial integration, enhancing the ability to capture detailed changes and supporting the personalization of rehabilitation treatments.

In this context, ultrasound-based techniques have evolved from purely diagnostic to functionally informative and quantitative modalities, attempting to bridge the gap between research and clinical application at the bedside. At the muscle level, elastography and QUS provide measurable indices of stiffness, spasticity, and echogenicity, supporting clinical decision-making and treatment planning. Despite their promise, these approaches still present methodological heterogeneity and lack standardized acquisition and analysis protocols [6,103]. In contrast, TCD is already used in stroke clinical pathways for the evaluation of cerebral hemodynamics, and can also be used in the rehabilitation field, both for functional monitoring of the main cerebral arteries and in neurofeedback paradigms [9,89,104].

These tools can offer unique advantages in tracking disease progression, evaluating treatment outcomes, and could soon become pivotal in shaping personalized stroke rehabilitation strategies. The combination of advanced hemodynamic monitoring with innovative technologies has the potential to transform post-stroke assessment and rehabilitation, enabling increasingly personalized and evidence-based interventions. However, to foster widespread clinical adoption, it will be essential to promote the standardization of ultrasound protocols to ensure comparability of results across centers and populations. At the same time, the integration of artificial intelligence algorithms based on machine learning techniques represents a promising development direction: recent studies have shown that such approaches can improve the automatic analysis of Doppler signals, more accurately detect CBF abnormalities and support diagnosis and functional monitoring [105,106].

Future research should focus on the clinical application of ultrasound as a tool for assessing the post-stroke rehabilitation process, including the definition of reference values and, above all, the development of shared and standardized protocols. Furthermore, the integration of artificial intelligence and automated signal and image analysis techniques could increase the objectivity and reproducibility of measurements, favoring multicenter validation and wider adoption in clinical practice [107].

## Figures and Tables

**Table 1:** Rehabilitation modalities for stroke recovery.

Category	Technique	Features	Regulatory Status/ Current Utilization
Clinical treatment protocols	Constraint-Induced Movement Therapy (CIMT)	CIMT coupled with supplementary therapies yields heightened upper limb function in post-stroke subjects [20].	Approved/Clinical
	Action Observation Treatment (AOT)	Approach based on the role of the mirror neuron system in motor learning [21].	Approved/Clinical
	Mirror therapy	Implementation of mirror-symmetric movements as a form of motor priming [22].	Approved/Clinical
	Motor imagery	Mental rehearsal of movements to promote neuroplastic changes [23].	Approved/Clinical
	Biofeedback	Enhances awareness and control over physiological functions [24].	Approved/Clinical
Instrumental treatment techniques	Deep Brain Stimulation (DBS)	Targets specific brain regions for functional improvement [25].	Experimental
	Transcranial Direct Current Stimulation (tDCS)	Application of contralaterally controlled functional electrical stimulation [26].	Experimental
	repetitive Transcranial Magnetic Stimulation (rTMS)	Targeted transcranial magnetic stimulation on ischemic cortical regions [27].	Experimental
	Transcranial Ultrasound Stimulation (TUS)	Promotes neuroplasticity, thrombus dissolution, and functional recovery through mechanical and neurotrophic effects [28,29]	Early clinical phase/Preclinical
Robotic tools	Robot-assisted therapy	Utilization of robotic tools for training [30,31]; effective for improving strength and increasing patient engagement time.	Approved/Clinical
	Virtual reality and exoskeleton	Fusion of robotic and virtual reality tools for rehabilitation [32].	Approved/Clinical
Telemedicine systems	Telerehabilitation: home-based exergaming interventions	Deployment of exergames for training purposes [33]; extends treatment time and supports long-term monitoring.	Approved/Clinical
	Telerehabilitation: immersive virtual reality	Utilization of augmented or virtual reality for training [34]; supports remote engagement and extended rehabilitation.	Approved/Expanding clinical adoption

## Supplementary Materials

The following supporting information can be downloaded at: https://www.mdpi.com/article/10.3390/biomedicines13112594/s1, S1: Assessment Scales for Stroke Evaluation. References [108,109,110,111,112,113,114,115,116,117,118,119,120,121,122,123,124,125,126,127,128,129,130,131,132,133,134,135,136,137,138,139,140,141,142,143,144,145,146,147,148,149,150,151,152,153,154,155] are cited in Supplementary Materials.

## Abbreviations

The following abbreviations are used in this manuscript:

ADL	Activities of Daily Living
AI	Artificial Intelligence
AOT	Action Observation Treatment
BI	Barthel Index
CBF	Cerebral Blood Flow
CIMT	Constraint-Induced Movement Therapy
DBS	Deep Brain Stimulation
DIF	Differential Item Functioning
EEG	Electroencephalography
EMG	Electromyography
ETC	Cognitive Therapeutic Exercise
fMRI	Functional Magnetic Resonance Imaging
fNIRS	Functional Near-Infrared Spectroscopy
fTCD	Functional Transcranial Doppler
FIM	Functional Independence Measure
ICF	Classification of Functioning, Disability, and Health
ML	Machine Learning
PNF	Proprioceptive Neuromuscular Facilitation
QUS	Quantitative Ultrasound
rTMS	Repetitive Transcranial Magnetic Stimulation
TCD	Transcranial Doppler
tDCS	Transcranial Direct Current Stimulation
TMS	Transcranial Magnetic Stimulation
TUS	Transcranial Ultrasound Stimulation
US	Ultrasound
WHO	World Health Organization
