# Supplementary material for: From Qualitative to Quantitative Functional Assessment in Stroke Rehabilitation with a Focus on Ultrasound Role
Source: Biomedicines. 2025 Oct 23;13(11):2594. doi: 10.3390/biomedicines13112594 (PMC12649935; doi:10.3390/biomedicines13112594)
Supplement: Supplementary file 1 [file biomedicines-13-02594-s001.zip › biomedicines-3896633-supplementary.pdf]

Supplementary Materials

S1: Assessment Scales for Stroke Evaluation

It is noteworthy that assessment scales related to contextual factors, namely environmental and personal factors, are not available but are referred to the medical staff evaluation. The table below incorporates the following dimensions: Overall, Body and Structures, Sensory Functions, Cognitive Functions, Quality of Life, and Activities of Daily Living. Specifically, scales grouped under "General" are designed for providing a standardized assessment of the severity and extent of the stroke effects on the patient, offering a comprehensive overview of their conditions. In "Body and Structures," the anatomical and physiological condition of the body and its functioning (muscle strength, spasticity, balance and posture, motor function of limbs) are examined. The "Functions" are further divided into sensory and cognitive categories. The first one includes specific assessments of sensitivity, discrimination, and sensory perception in various modalities. The second one encompasses scales that allow the evaluation of a broad range of cognitive functions, from memory and attention to cognitive flexibility and language proficiency. "Quality of Life" encompasses all tools that assess the patient's well-being and perceptions across various aspects of life, including physical health, mental health, and social relationships. "Activities of Daily Living" scales aid in understanding the patient's autonomy in daily life, specifically their level of functional independence in essential everyday activities.

**Overview**	**Scale**		**Assessments**
**GENERAL**	NIHSS	National Institute of Health Stroke Scale	Neurological function in various domains, such as consciousness, muscle strength, and coordination [108].
	CNS	Canadian Neurological Scale	Severity of a stroke, focusing on parameters such as consciousness, muscle strength, and reflexes [109].
	ESS	European Stroke Scale	Various neurological functions, including strength, coordination, and sensitivity [110].
	SSS	Scandinavian Stroke Scale	Severity of the stroke, with an emphasis on neurological function, including aspects such as muscle strength and sensitivity [111].
	SIAS	Stroke Impairment Assessment Set	Extent of disabilities caused by a stroke, including assessments of mobility and motor function [112].
**BODY AND STRUCTURE**	FMA	Fugl-Meyer Assessment	Post-stroke motor recovery, focusing on strength, coordination, and motor function [113].
	MI	Motricity Index	Muscle strength in patients with neurological disabilities, focusing on the arm, hand, and leg [114].
		Modified Ashworth spasticity scale	Muscle tone and spasticity in patients with central nervous system injuries, such as those caused by strokes [115].
	MAS	Motor Assessment Scale	Motor functional capacity in patients with neurological disabilities [116].
	FTHUE	Functional Test for the Hemiplegic Upper Extremity	Motor function of the upper limbs in individuals affected by hemiplegia [117].
		Berg Balance Scale	Balance and stability in individuals with disabilities, including those post-strokes [118].
	MUSUPES	Motor Evaluation Scale for Upper Extremity in Stroke Patients	Motor function, focusing on muscle tone, movement quality, and the ability to perform specific arm and hand tasks [119].

	STREAM	Stroke Rehabilitation Assessment of Movement	Movement and motor function in post-stroke patients [120].
		Chedoke-McMaster Stroke Assessment	Motor and functional recovery in post-stroke patients, with a particular focus on the lower limbs [121].
	FDA	Frenchay Dysarthria Assessment	Joint and voice disorders in individuals with neurological disabilities [122].
	RMI	Rivermead Mobility Index	Mobility and independence in walking, critical aspects in post-stroke rehabilitation [123].
	FAT	Frenchay Arm Test	Motor function of the upper limb [124].
	MAL	Motor Activity Log	Use of the upper limb in daily life [125].
**SENSORY FUNCTIONS**		Nottingham Sensory Assessment	Touch sensitivity, tactile discrimination, and thermal sensitivity [126].
		Two-Point Discrimination Test	Tactile discrimination by determining the minimum distance at which a patient can perceive two distinct points [127].
	SOT	Sensory Organization Test	Nervous system's ability to use sensory input to maintain balance [128].
	VAS	Visual Analog Scale	Pain perception, allowing patients to indicate their pain experience on a scale [129].
		Fugl-Meyer Assessment (Sensory Part)	Sensitivity and sensory perception in the extremities [113].
	FRT	Functional Reach Test	Ability to reach forward by measuring the maximum distance a person can reach while standing [130].
	TTDPM	Threshold to Detection of Passive Motion	Ability to detect passive joint movement, providing information about movement sensitivity [131].
	JPR	Joint Position Reproduction	Patient's ability to accurately reproduce the position of a joint, measuring proprioceptive sensitivity [132].
	AMEDA	Active Movement Extent Discrimination Assessment	Accuracy in discerning active movement extension [133].
**COGNITIVE FUNCTIONS**	MoCA	Montreal Cognitive Assessment	Cognitive functions, including attention, memory, and executive abilities [134].
	MMSE	Mini-Mental State Examination	Cognitive function, including questions about memory, attention, language skills, and orientation [135].
	CDT	Clock Drawing Test	Requires the patient to draw a clock to assess executive and visuospatial functions [136].
	TMT	Trail Making Test	Processing speed, cognitive flexibility, and visuospatial attention [137].
	ACE	Addenbrooke's Cognitive Examination	Various cognitive areas, including executive functions, memory, and language [138].
	SUMSE	Stroke Unit Mental Status Examination	Mental state of stroke-affected patients, focusing on aspects such as attention, memory, and language [139].
	VF	Verbal Fluency Test	Linguistic function and executive abilities [140].

	BNT	Boston Naming Test	Linguistic function and semantic memory [141]
		Token Test	Auditory comprehension and language processing [142].
**QUALITY OF LIFE**	SIS	Stroke Impact Scale	Quality of life across different dimensions, including mobility, cognitive function, independence, and social participation [143].
	QLI-Generic Version	Ferrans & Powers Quality of Life Index	Quality of life considering physical, psychological, relational, and economic aspects [144].
	SF-36	36-Item Short-Form Survey	Quality of life across various dimensions, including physical and mental health. Applicable to a variety of medical conditions, including post-stroke patients [145].
	EQ-5D	EuroQol Five-Dimension Scale	Health-related quality of life across five dimensions: mobility, self-care, usual activities, pain/discomfort, and anxiety/depression [146].
	SS-QOL	Stroke-Specific Quality of Life Scale	Quality of life through specific questions related to post-stroke effects, such as communication, mobility, and social participation [147].
	HDRS	Hamilton Depression Rating Scale	Severity of depression, addressing symptoms such as mood, insomnia, appetite, guilt, agitation, anxiety, and suicidal thoughts [148].
**ACTIVITIES OF DAILY LIVING**	BI / MBI	Barthel Index / Modified Barthel Scale	Independence in basic daily activities (e.g., eating, dressing, and showering) [40]. The MBI has been adapted to be more sensitive to changes in disability, allowing for better detection of improvement or worsening [149].
	FIM	Functional Independence Measure	Patient's level of functional independence in various activities of daily living, considering both basic and advanced activities [45].
	ADL	Katz Index of Independence in Activities of Daily Living	Independence in basic activities [150].
	IADL	Lawton-Brody Instrumental Activities of Daily Living Scale	Instrumental activities of daily living, such as managing finances, meal preparation, and phone use [151].
	OTTIAQ	Occupational Therapy Task-Oriented Approach	Independence in daily activities through goal-oriented specific tasks [152].
	COPM	Canadian Occupational Performance Measure	Satisfaction and effectiveness in performing specific daily activities, providing a patient-centered perspective [153].
	mRS	Modified Rankin Scale	Degree of disability or functional dependence in patients [154].
	PSMS	Physical Self-Maintenance Scale	Independence in physical self-maintenance activities (eating, dressing, and toileting) [155].
